# Genetics in Osteoarthritis

**DOI:** 10.2174/138920208786847953

**Published:** 2008-12

**Authors:** Mercedes Fernández-Moreno, Ignacio Rego, Vanessa Carreira-Garcia, Francisco J Blanco

**Affiliations:** Osteoarticular and Aging Research Lab, Genomics Unit. Biomedical Research Center, INIBIC-CH Universitario A Coruña, A Coruña, Spain

**Keywords:** Osteoarthritis, genetics, chondrocyte, cytokines.

## Abstract

Osteoarthritis is a degenerative articular disease with complex pathogeny because diverse factors interact causing a process of deterioration of the cartilage. Despite the multifactorial nature of this pathology, from the 50’s it´s known that certain forms of osteoarthritis are related to a strong genetic component. The genetic bases of this disease do not follow the typical patterns of mendelian inheritance and probably they are related to alterations in multiple genes. The identification of a high number of candidate genes to confer susceptibility to the development of the osteoarthritis shows the complex nature of this disease. At the moment, the genetic mechanisms of this disease are not known, however, which seems clear is that expression levels of several genes are altered, and that the inheritance will become a substantial factor in future considerations of diagnosis and treatment of the osteoarthritis.

## INTRODUCTION

Osteoarthritis is a degenerative articular disease with a complex pathogeny because diverse factors interact causing a process of deterioration of the cartilage and the subchondral bone. It can be primary or secondary to diverse diseases, but it has clinical, radiological, and pathological manifestations in common. Its pathogenesis is complex due to genetic, metabolic and local factors, which interact and cause a process of deterioration of the cartilage, with a proliferative reaction of subchondral bone and synovial inflammation. Apart from the classical concept of disease being age related, osteoarthritis is considered as a heterogeneous group, with common and different aspects; its diagnosis is variable with real possible treatments.

Chondrocytes must play an important role in the early stages, and in the progression and development of the disease [1]. Despite this, there are various factors that have a relevant role in the development of the disease, such as age, sex, race, lifestyle, obesity, occupation or genetics [2]. Since the 1950s, it´s known that certain types of osteoarthritis are related with a strong genetic component.

The first study developed in this sense was carried out by Stechner in 1941 [3], who demonstrated that the presence of Heberden nodules in osteoarthritic fingers on the hand were three times more likely to occur in twins compared to the general population. It was concluded that these lesions were inherited as an autosomal dominant character, with an elevated prevalence in women. Similar family studies performed in the 1960s in the United Kingdom in individuals that had symptoms of general osteoarthritis showed, as in the previous case, that there must be a relationship between the disease and being a twin sister [4]. Post studies support the existence of family ties in relation to suffering Heberden or Bouchard nodules [5-7].

The genetics of this disease is complex, as it does not usually follow the typical pattern of mendelian inheritance and it is probably associated with multiple gene interactions. A large number of studies support the theory of a polygenic inheritance, as opposed to defect in a single gene [8,9]. The beginnings, progression and severity of the disease may be influenced by the development of multiple gene factors, which interact with diverse alterations produced by different genes. The genetic influence of this disease is estimated between 35% and 65% [10,11]. Epidemiological studies estimate that there is a 40% probability of inheritability in an osteoarthritic knee and a 65% probability of inheritability in osteoarthritic hands and hips [12].

The identification of the loci related with osteoarthritis in both hands and other articulations suggested that genes being susceptible to developing osteoarthritis are not specific to the articulation, which is coherent with the hypothesis “common variants-multiple disease”, characteristic of complex genetic disorders [13].

### Identification of Candidate Genes that Confer Susceptibility of Developing Osteoarthritis

Two approximations have traditionally been considered when identifying genes which confer susceptibility to developing a complex pathology: genome wide linkage scan and study of candidate genes.

### Genome Wide Linkage Scan

This procedure does not require any previous knowledge of the nature or function of the gene, since it is a genomic scan searching for genes associated in some way with the pathology. It is an expensive technique, which requires the genotyping of various polymorphic markers from a large number of affected people belonging to the same family. Through the development of this technique, the relationships between 12 chromosomes (1, 2, 4, 6, 7, 9, 11-13, 16, 19 and X) and this disease have been detected, showing the complexity of the transmission of osteoarthritis. In distinct analysis carried out on chromosomes 2p, 2q, 7p, 11q and 16p, five loci were detected; these chromosomes present a greater probability of being gene carriers involved in the osteoarthritic process [14-19].

### Study of Candidate Genes

Study of candidate genes is orientated towards the search for alterations in a gene, which requires knowledge in its function and the possible pathological role. Some genes related with the osteoarthritic process are shown in Table **1**.

#### Genes that Code for Collagen

Among the candidate genes that play a role in the osteoarthritic process, the genes that code for structural proteins of the extracellular matrix of the cartilage seem to have an important role, especially those that code for collagen type II (COL2A1). There are many reasons to suggest that alterations in this gene are responsible for the degeneration of the joints during the osteoarthritic process, such as the association of diverse mutations with distinct osteochondrodysplasia or the proper function of COL2A1, that codes for the most abundant proteins in the extracellular matrix [10,18,20]. Among the remaining genes that code for the structural proteins of the extracellular matrix, some candidates present an increased susceptibility to developing the disease, including COL9A2, COL11A1, COL11A2 [21, 22, 23] or COMP (cartilage oligomeric matriz protein gene) [24]. On the other hand, some studies suggest a protective role that could cause alterations in these genes. As shown in the study by Lian et al. (2005) [25], they concluded that there is an association between certain mutations in the COL1A1 gene and the decreased probability of suffering hip osteoarthritis in women. However, not all of the published studies support the conclusion that the candidate genes increase the susceptibility to developing the disease [26,27].

#### Interleukines

Recent studies have demonstrated the importance of the inflammatory process in the pathology of osteoarthritis [28-33]. Even though this disease is not a classic autoimmune process, various cytokines are involved in the metabolism of the cartilage and are synthesized not only by synovial cells but also by cartilage chondrocytes. Interleukin 1 (IL-1) is the main catabolic cytokine that exists in the articulation and it stimulates the synthesis of a number of proteinases, which can simultaneously destroy the extracellular cartilage. When the anabolic and catabolic activities of cytokines are in balance, cartilage integrity is maintained. If there is a factor that favours catabolism then a degenerative process within the cartilage will occur, resulting in a final osteoarthritic process [34]. Therefore, it is reasonable to suggest that various genes, which code for different interleukins [35], may influence genetic susceptibility to osteoarthritis. Genes coding for IL-1A, IL-1B, IL1RN, IL4R, IL17A, IL17F and IL6 were analyzed and various alterations in these regions of DNA, including SNPs (single nucleotide polymorphisms), microsatellites and VNTR (variable number tandem repeats) has been described and related with osteoarthritis in different articulations [36-40].

#### Other Genes Implicated in the Osteoarthritic Process

The estrogen receptor α gene (ERα) is an important mediator in signal transduction, and is expressed in different cells, including human chondrocytes [41, 42]. Numerous alterations in this gene have shown to affect the structure and the function of this protein, which as a last resort can affect the progression of osteoarthritis [43]. Epidemiological studies carried out in women, suggest that the loss of estrogen may be accompanied by an increase in the prevalence of hip and knee osteoarthritis [44], which would help to explain the differences in the prevalence of this disease with respect to gender.

Vitamin D and its receptor (VDR) play an important role in bone metabolism, in response to the immune system, cancer and osteoarthritis [45]. VDR has an important function in regulating calcium metabolism and cellular function in bones [46]. Diverse studies proved the relationship between the levels of vitamin D in serum and the progression of osteoarthritis in the knee [47]. Meanwhile others reflect the relationship between certain polymorphisms in the gene that codes for vitamin D and the susceptibility of developing osteoarthritis in different joints [48-50].

The Frizzled Related Protein gene (FRZB), which codes for the secretions of FRZB (FRZB3), is involved in both the bone formation and the negative regulation of the receptor-signalling pathway Wnt, whose inhibition is important in maintaining the structure of the cartilage [51]. Different mutations in this gene are related with the development of osteoarthritis [52].

Asporins (ASP), characterized for the first time by Lorenzo* et al. *(2001) [53], are components of the extracellular matrix and expressed in high proportions in cartilage of osteoarthritic patients. They act as negative regulators of chondrogenesis, inhibiting the action of TGFβ by direct contact [54].

Apart from the genes described above, there has also been an increase in projects over the past few years that have contributed to the discovery of new genes related to osteoarthritis. The ones that stand out are genes that code for structural proteins and proteins related to the loss of cartilage, as well as genes related to the increase in synthesis of the extracellular matrix and with an adaptive response to the disintegration of cartilage. We can mention aggrecan (AGC1), insulin-like growth factor (IGF-1), transforming growth factor β (TGFβ1), tissue inhibitor of metalloproteinase 3 (TIMP3), the metalloprotease gene ADAM12, leucine-rich repeats and calponin homology domain containing 1 gene LRCH1, the calmodulin gene (CALM1), Matrilin 3 (MATN-3), CLIP (cartilage intermediate protein), TNA (tetranectin), and BMP2 (bone morphogenic protein 2) [1,17, 55-59]. 

### Cases of Non-Primary Osteoarthritis

Several osteoarthritis are related to alterations in HLA-A1B8 [60], certain haplotypes of HLA-B8 [61] and with diverse isoforms of α1 antitrypsin [60], however not all studies show the same association [62]. In the same way different types of rare osteoarthritis exist whose presence are related to diverse pathologies, such as the family of calcium pyrophosphate deposits disease (CPDD) [63,64], Stickler syndrome [65] and some chondrodysplasias [66], which present a clear genetic base.

### Expression Libraries and Microarray Technology

In all the studies carried out up until now, sufficient evidences exist to confirm that the chondrocytes play an important role in cartilage degradation. A change in the gene expression levels of patients with this disease is expected as a response to both exogenous and endogenous stimuli. Based on this, different groups studied the genetic expression obtaining different responses in function to the region of cartilage analyzed. Kumar *et al*. (2001) [67] obtained the first cDNA library of cartilage from osteoarthritic and normal patients. After analyzing 10,000 EST (expressed sequence tags) two new homologous proteins that are well known were obtained [procollagen C-proteinase enhnacer protein-2 (PCPE-2) and GalNAC transferase], and they also described genes which had never been described in cartilage before. Futher studies in this field were undertaken and in 2006 the number of ESTs described reached ~ 117,000 [68] and the development of the transcription map in cartilage is estimated to contain 16,000 genes.

Since the mid 90s the EST, clones and cDNA databases have contributed to important developments in microarray technology. At the beginning of the year 2000 investigations into genomic functions of the cartilage were developed, so Aigner *et al*. (2001) [69] published the first study of microarrays in osteoarthritis. Sato *et al*. (2006) [70] used Affymetrix GeneChip^®^technology and analyzed the gene expression profile of cartilage from knees of osteoarthritic patients, from both injured and healthy regions. They identified different expression levels of transcripts with respect to the origins of the sample. In the same study, expression levels in osteoarthritic and healthy individuals were compared.

### Mitochondrial Genetics in OA

Mitochondria are organelles present in most eukaryotic cells and play a crucial role in ATP synthesis by oxidative phosporilation (OXPHOS); furthermore, heat production, reactive oxygen species (ROS) generation, apoptosis and several metabolic pathways are some aspects in which these organelles are involved. Despite the glycolytic nature of articular chondrocytes, the mitochondrially-mediated pathogenesis of OA is being increasingly investigated [71-73]. Besides, given the role that the proteins encoded by the mitochondrial genome play in the production of ATP, mutations of mitochondrial DNA (mtDNA) are an important and common cause of human disease, affecting approximately one out every 3500 individuals [74].

The evolution of human mtDNA is characterized by the emergence of distinct maternal lineages that defined haplogroups characterized by specific mtDNA polymorphisms [75]. Among Europeans, 95% of the population belongs to one of nine haplogroups: H, I, J, K, T, U, V, W or X. Since mitochondrial dysfunction and defective oxidative phosphorylation have been linked to some human disorders [76], and given the lines of evidence that describe the contribution of mtDNA to cellular physiology and its critical importance for energy production, there are a number of studies investigating the association between mtDNA haplogroups and multifactorial diseases [77] and aging [78]. Our group, in a recent study carried out in 457 knee OA subjects and 262 radiological healthy controls from an Spanish population, demonstrated that European mtDNA haplogroup J is involved in conferring protection from incidence (OR = 0.460; 95% CI: 0.282 – 0.748; p = 0.002) and severity (OR = 0.351; 95% CI: 0.156 – 0.787; p = 0.012) of knee OA, while mtDNA haplogroup U appears to be associated with an increase in knee OA radiological grade severity (OR = 1.788; 95% CI: 1.094 – 2.922; p = 0.025), being the first study that correlates mtDNA haplogroups with OA [79].

## CONCLUSIONS

As described above, osteoarthritis is a complex disease that not only involves the cartilage; the joint and its interaction with the cartilage must also be considered. Even though the genetic mechanisms of this disease are not actually known, it seems clear that expression levels in osteoarthritic and healthy patients are different and that genetic inheritance is a factor that should be considered in the near future for diagnosing and treating osteoarthritis. On the other hand, it is important to recognize the interaction between genetic and environmental factors (e.g. obesity, excessive stress on the joint articulation, or the type of occupation) as it can be critical in the clinical expression of the disease. Advances over the last few years in the genetic knowledge of osteoarthritis has allowed us to be optimistic over the early detection of this disease, as well as the identification of possible osteoarthritic subpopulations and the ability to forecast a response when treated with different therapies.

## Figures and Tables

**Table 1:** Genes Related with the Osteoarthritic Process

Gene	Phenotypic Manifestation	Reference
COL11A1	Early apparition of osteoarthritis	[21, 23]
COL11A2	Early apparition of osteoarthritis	[22]
COL1A1	Reduction in osteoarthritis in female hips	[26]
COL2A1	Early apparition of osteoarthritis	[20, 26, 59]
COL9A1	Early apparition of knee osteoarthritis	[18, 27]
COMP (cartilage oligomeric matrix protein gene)	Early apparition of hip osteoarthritis	[24, 59]
Interleukin 1 (IL-1A, IL-1B, IL1RN)	Knee and hip osteoarthritis	[27, 38]
Interleukin 6	Hip osteoarthritis	[40]
Interleukin 17 (IL17A, IL17F)	Susceptibility to developing osteoarthritis	[39]
Interleukin 4 (IL4R)	Hip osteoarthritis in females	[37]
Oestrogen α receptor (Erα)	Osteoarthritis in women	[43, 44]
Vitamin D receptor (VDR)	Osteoarthritis in various joints	[48, 49, 50]
Frizzled Related Protein (FRZB)	Hip osteoarthritis in females	[51, 52, 59]
Asporin (ASP)	Knee and hip osteoarthritis	[53, 54, 59]
Aggrecans (AGC1)	Hand osteoarthritis	[1]
Insulin-like growth factor 1 (IGF-1)	Increased risk in developing osteoarthritis	[1, 20, 55]
Transforming growth factor β (TGFβ1)	Osteoarthritis	[1]
Tissue inhibitor of metaloprotease 3 (TIMP3)	Knee and hip osteoarthritis	[1]
Metaloprotease ADAM12	Knee osteoarthritis	[58]
Repetitions rich in leukine	Knee osteoarthritis	[17, 57]
Calponin homology domain containing protein 1 (LRCH1)	Knee osteoarthritis	[17, 57]
Calmodulin (CALM1)	Hip osteoarthritis in Japanese population	[59]
Matrilin-3	Early apparition of osteoarthritis in knees and hands	[56]
Cartilage intermediate protein (CLIP)	Knee osteoarthritis in males	[58]
Tetranectin (TNA)	Knee osteoarthritis	[58]
Bone morphogenic protein 2 (BMP2)	Reduction in osteoarthritis in female knees	[58]

## References

[1] Aigner T, Dudhia J (2003). Genomics of osteoarthrosis. Curr Opin Rheumatol.

[2] Felson DT, Zhang Y (1998). An update on the epidemiology of knee and hip osteoarthritis with a view to prevention. Arthritis Rheum.

[3] Stecher RM (1941). Herberden´s nodes. Heredity in hypertrophic arthritis of the finger joints. Am J Med Sci.

[4] Kellgren JH, Lawrence JS, Bier F (1963). Genetic factors in generalised osteoarthritis. Ann Rheum Dis.

[5] Allison AC, Blumberg BS (1953). Familiar osteoarthropathy of the fingers. J. Bone Join Surg. Br.

[6] Buchana WW, Park WM (1983). Primary generalized osteoarthritis: definition and uniformity. J. Rheumatol.

[7] Crain DC (1961). Interphalangeal osteoarthritis characterized by painful inflammatory episodes resulting in deformity of the proximal and distal articulations. JAMA.

[8] Lawrence JS (1977). Rheumatism in population.

[9] Lawrence JS, Gelsthorpe K, Morell G (1983). Heberden´s nodes and HLA markers in generalised osteoarthritis. J. Rhuematol. Suppl.

[10] Cicuttini FM, Spector TD (1996). The genetics of osteoarthritis. J. Clin. Pathol.

[11] Cicuttini FM, Spector TD (1996). Genetics of osteoarthritis. Ann. Rheum. Dis.

[12] Spector TD, McGregor AJ (2004). Risk factors for osteoarthritis. Osteoarthritis Cartilage.

[13] Greig G, Spreckley K, Aspinwall R, Gillaspy E, Grant M, Ollier W, Jonh S, Doherty M, Wallis G (2006). Linkage to nodal osteoarthritis: quantitative and qualitative analyses of data from a whole-genome screen idetify trait-dependent susceptibility *loci*. Ann. Rheumatol. Dis.

[14] Demissie S, Cupples LA, Myers R, Aliabadi P, Levy D, Felson DT (2002). Genome scan for quantity of hand osteoarthritis. The Framinghan study. Arthritis Rheum.

[15] Loughlin J (2001). Genetic epidemiology of primary osteoarthritis. Curr. Opin. Rheumatol.

[16] Loughlin J, Dowling B, Mustafa Z, Southam L, Chapman K (2002). Refined linkage mapping of a hip osteoarthritis susceptibility locus on chromosome 2q. Rheumatology.

[17] Loughlin J (2006). Osteoarthritis linkage scan: more *loci* for the geneticist to investigate. Ann. Rheumatol. Dis.

[18] Mustafa Z, Chapman K, Irven C, Carr AJ, Clipsham K, Chitnavis J, Sinsheimer JS, Bloomfield VA, McCartney M, Cox O, Sykes B, Loughlin J (2000). Linkage analysis of candidate genes as susceptibility *loci* for osteoarthritis-suggestive linkage of COL9A1 to female hip osteoarthritis. Rheumatology.

[19] Ingvarsson T, Stefansson SE, Gulcher JR, Jonson HH, Jonson H, Frigge ML, Pálsdóttir E, Olafsdóttir G, Jónsdóttir T, Walters GB, Lohmander LS, Stefánsson K (2001). A large Icelandic family with early osteoarthritis of the hip associated with a susceptibility locus on chromosome 16p. Arthritis Rheum.

[20] Zhai G, Rivadeneira F, Houwing-Duistermaat J, Meulenbelt I, Bijkerk C, Hofman A, Van Meurs JB, Uitterlinden AG, Pols HA, Slagboom PE, van Duijn CM (2004). Insulin-like growth factor I gene promoter polymorphims, collegen type II α1 (COL2A1) gene, and the prevalence of radiographic osteoarthritis: the Rotterdam study. Ann. Rheumatol.

[21] Holden P, Canty EG, Mortier GR, Zabel B, Spranger J, Carr A, Grant ME, Loughlin JA, Briggs MD (1999). Identification of novel pro-α2 (IX) collagen gene mutations in two families with distinctive oligo-epiphyseal froms of multiple epiphyseal dysplasia. Am. J. Hum. Genet.

[22] Vikkula M, Mariman EC, Lui VC, Zhidkova NI, Tiller GE, Goldring MB, van Beersum SE, de Waal Malefijt MC, van den Hoogen FH, Ropers HH, Mayne R, Cheah KS, Olsen BR, Warman ML, Brunner HG (1995). Autosomal dominant and recessive osteochondrodysplasias associated with the COL11A2 locus. Cell.

[23] Richards AJ, Yates JR, Williams R, Payne SJ, Pope FM, Scott JD, Snead MP (1996). A family with Strickler syndrome type 2 has a mutation in the COL11A1 gene resulting in the substitution of glycine 97 by valine in α1 (XI) collagen. Hum. Mol. Genet.

[24] Loughlin J, Irven C, Mustafa Z, Briggs MD, Carr A, Lynch SA, Knowlton RG, Cohn DH, Sykes B (1998). Identification of five novel mutations in the cartilage oligomeric matirx protein gene in pseudoachondroplasia and multiple epiphyseal dysplasia. Hum. Mutat.

[25] Lian K, Zmuda JM, Nevitt MC, Lui L, Hochberg MC, Greene D, Li J, Wang J, Lane NE (2005). Type I collagen alpha 1 Sp1 transcription factor binding site polymorphism is associated with reduced risk of hip osteoarthritis defined by severe joint space narrowing in elderly women. Arthritis Rheum.

[26] Loughlin J, Irven C, Fergusson C, Sykes B (1994). Sibling pair analysis shows no linkage of generalized osteoathritis to the *loci* encoding type II collagen, cartlige link protein or cartilage matrix protein. Br. J. Rheum.

[27] Loughlin J, Mustafa Z, Dowling B, Southam L, Marcelline L, Räinä SS, Ala-Kokko L, Chapman K (2002). Fine linkage mapping of a primary hip osteoarthritis susceptibility locus on chromosome 6. Eur. J. Hum. Genet.

[28] Pelletier JP, Johanne MP, Abramson SB (2001). Osteoartrhitis an inflamatory diseasse: potencial implication for the selection of new therapeutic targets. Arthritis Rheum.

[29] Yuan GH, Kayo MH, Kato T, Nishioka K (2003). Immunologic intervention in the pathogenesis of osteoarthritis. Arthritis Rheum.

[30] Sandell LJ, Aigner T (2001). Articular cartilage and changes in arthritis. An introducction cell biology of osteoarthritis. Arthritis Res.

[31] Lotz M, Blanco F, von Kempis J, Dudler J, Maier R, Villiger PM, Geng Y (1995). Cytokine Regulation of Chondrocyte Functions. J. Rheumatol.

[32] Attur MG , Dave M , Akamatsu M , Katoh M , Amin AR  (2002). Osteoarthritis or osteoarthrosis: the definition of inflammation becomes a semantic issue in the genomic era of molecular medicine. Osteoarthritis Cartilage.

[33] Sakkas LI, Platsoucas CD (2007). The role of T cells in the pathogenesis of Osteoarthritis. Arthritis Rheum.

[34] Blanco FJ (1999). Catabolic events in Osteoarthritic Cartilage. Ostearthritis Cartilage.

[35] Blanco FJ, Lotz M (1995). Citoquinas, condrocitos y artrosis. Rev. Esp. Reumatol.

[36] Loughlin J, Dowling B, Mustafa Z, Chapman K (2002). Association of the Interleukin-1 gene cluster on chromosome 2q13 with knee osteoarthritis. Arthritis Rheum.

[37] Foster T, Chapman K, Loughlin J (2004). Common variants within the interleukin 4 receptor α gene (IL4R) are associated with susceptibility to osteoarthritis. Hum. Genet.

[38] Meulenbert I, Seymour AB, Nieuwland M, Huizonga TWI, van Dujin CM, Slagboom PE (2004). Association of the Interleukin-1 gene cluster with radiographic signs of osteoarthritis of the hip. Arthritis Rheum.

[39] Southam L, Heath O, Chapman K, Loughlin J (2006). Association analysis of the interleukin 17 genes IL17A and IL17F as potential osteoarthritis susceptibility *loci*. Ann. Rheumatol. Dis.

[40] Pola E, Papaleo P, Pola R, Gaetanis E, Tamburelli FC, Aulisa L, Logroscino CA (2005). Interleukin-6 gene polymorphism and risk of osteoarthritis of the hip: a case-control study. Osteoarthritis Cartilage.

[41] Tsai CL, Liu TK, Chen TJ (1992). Estrogen and osteoarthritis: a study of synovial estradiol and estradiol receptor binding in uman osteoarthritis knees. Biochem. Biophys. Res. Commun.

[42] Richmond RS, Carlson CS, Register TC, Shnaker G, Loeser RF (2000). Functional estrogen receptor in adult articular cartilage: estrogen replacement therapy increase chondrocytes syntesis of proteoglycans and insulin-like growth factor binding protein 2. Arthritis Rheum.

[43] Bergink AP, van Meurs JB, Loughlin J, Arp PP, Fang Y, Hofman A, van Leeuwen JP, van Duijn CM, Uitterlinden AG, Pols HA (2003). Estrogen receptor α gene haplotipe is associated with radiographic osteoarthritis of the knee in elderly men and woman. Arthritis Rheum.

[44] Richette P, Corvol M, Bardin T (2003). Estrogens, cartilage, and osteoarthritis. Joint Bone Spine.

[45] Haussler MR, Whitfield GK, Haussler CA, Hsieh JC, Thompson PD, Selznick SH, Domínguez CE, Jurutka PW (1998). The nuclear vitamin D receptor: biological and molecular regulatory properties reveald. J. Bone Miner. Res.

[46] Uitterlinden AG, Fang Y, van Leeuwen H, Pols HA (2004). Vitamin D receptor gene polymorphisms in relation to vitamin D related diseas states. J. Steroid Biochem. Mol. Biol.

[47] McAlindon TE, Felson DT, Zhang Y, Hannan MT, Aliabadi P, Weissman B, Rush D, Wilson PW, Jacques P (1996). Relation of dietary intake and serum levels of vitamin D to progression of osteoarthritis and the knee among pasticipants in the Fram ingham Study. Ann. Intern. Med.

[48] Uitterlinder AG, Burger H, Huang Q, Odding E, van Duijn C, Hofman A, Birkenhäger JC, van Leeuwen JP, Pols HA (1997). Vitamin D receptor genotype is associated with radiographic osteoarthritis at the knee. J. Clin. Invest.

[49] Uitterlinder AG, Burger H, van Duijn C, Huang Q, Hofman A, Birkenhäger JC, van Leeuwen JP, Pols HA (2000). Adjacent genes, for COL2A1 and the vitamin D receptor, are associated with separate features of radiographic osteoarthritis of the knee. Arthirtis Rheum.

[50] Solovieva S, Hirvonen A, Siivola P, Vehmas T, Luoma K, Riihimäkii H, Leino-Arjas P (2006). Vitamin D receptor gene polymorphisms and susceptibility of hand osteoarthritis in Finnish women. Arthritis Res. Ther.

[51] Loughlin J, Dowling B, Chapman K, Marcelline L, Mustafa Z, Southam L, Ferreira A, Ciesielski C, Carson DA, Corr M (2004). Functional variants within the secreted frizzled-related protein 3 gene are associated with hip osteoarthritis in females. Proc. Natl. Acad. Sci. USA.

[52] Rodriguez-Lopez J, Pombo-Suarez M, Gomez-Reino JJ, Gonzalez A (2007). Further evidence of the role of frizzled-related protein gene polymorphisms in osteoarthritis. Ann. Rheumatol. Dis.

[53] Lorenzo P, Aspberg A, Onnerfjord P, Bayliss MT, Neame PJ, Heinegard D (2001). Identification and characterization of asporin, a novel menber of the leucine-rich repeat protein family closely related to dEcoRIn and biglycan. J. Biol. Chem.

[54] Kizawa H, Kou I, Iida A, Sudo A, Miyamoto Y, Fukuda A, Mabuchi A, Kotani A, Kawakami A, Yamamoto S, Uchida A, Nakamura K, Notoya K, Nakamura Y, Ikegawa S (2005). An aspartic acid repeat polymorphism in asporin inhibits chondrogenesis and increase susceptibility to osteoarthritis. Nat. Genet.

[55] Tyler JA (1989). Insulin-like growth factor 1 can decrease degradation and promote synthesis of proteoglycan in cartilage exposed to cytokine. Biochem. J.

[56] Stefánsson SE, Jónsson H, Ingvarsson T, Manolescu I, Jósson HJ, Olafsdóttir G, Pálsdóttir E, Stefánsdóttir G, Sveinbjörnsdóttir G, Frigge ML, Kong A, Gulcher JR, Stefánsson K (2003). Genomewide scan for hand osteoarthritis: a novel mutation in matrilin-3. An. J. Hum. Genet.

[57] Spector TD, Reneland RH, Mah S, Valdes AM, Hart DJ, Kammerer S, Langdown M, Hoyal CR, Atienza J, Doherty M, Rahman P, Nelson MR, Braun A (2006). Association between a variation in LRCH1 and knee osteoarthritis. Arthritis Rheum.

[58] Valdes AM, van Oene M, Hart DJ, Surdulescu GL, Loughlin J, Doherty M, Spector TD (2006). Reproducible genetic associations between candidate genes and clinical knee osteoarthritis in men and women. Arthritis Rheum.

[59] Valdes AM, Loughlin J, van Oene M, Chapman K, Surdulescu GL, Doherty M, Spector TD (2007). Sex and ethnic differences in the association of ASPN, CALM1, COL2A1, COMP, and FRZB with genetic susceptibility to osteoarthritis of the knee. Arthritis Rheum.

[60] Patrick M, Manhire A, Ward M, Doherty M (1989). HLA-AB antigens and α1-abtitrypsin phenotypes in nodal and generalized osteoarthritis and erosive osteoarthritis. Ann. Rheumatol. Dis.

[61] Brodsky T, Appelboam A, Govaerts Famey JP (1979). Antigens HLA et nodosites d´Heberden. Acta Rheumatol.

[62] Spector TD, Cicuttini F, Baker J, Loughlin J, Hart DJ (1996). Genetic influence on osteoarthritis: A twin study. BMJ.

[63] Ryan LM, McCarty DJ, McCarty DJ, Koopman WJ (1993). Calcium pyrophosphate cristal deposition disease: Pseudogout; articular chondrocalcinosis. Artritis and allied condition.

[64] Zitnam D, Sitaj S (1960). Chondrocalcinosis polyarticularis (familiars). Radiol. Diagn.

[65] Stickler GB, Belau PG, Farrell FJ, Hayles AC (1965). Hereditary progressive arthroophthalmopathy. Mayo Clin. Proc.

[66] Zitnam D, Sitaj S (1963). Chondrocalcinosis articularis Section L Clinical and radiological study. Ann. Rheumatol. Dis.

[67] Kumar S, Connor JR, Dodds RA, Halsey W, Van Horn M, Mao J, Sathe G, Mui P, Agarwal P, Badger AM, Lee JC, Gowen M, Lark MW (2001). Identification and initial characterization of 5000 expressed sequenced tags (ESTs) each from adult human normal and osteoarthritic cartilage cDNA libraries. Osteoarthritis Cartilage.

[68] Marshall KW, Zhang H, Nossova N (2006). Chondrocyte genomics: implications for disease modification in osteoarthritis. Drug Discov. Today.

[69] Aigner T, Zien A, Gehrsitz A, Gebhard PM, McKenna L (2001). Anabolic and catabolic gene expression pattern analysis in normal versus osteoarthritic cartilage using complementary DNA-arry technology. Arthritis Rheum.

[70] Sato T, Konomi K, Yamasaki S, Aratani S, Tsuchimochi K, Yokouchi M, Masuko-Hongo K, Yagishita N, Nakamura H, Komiya S, Beppu M, Aoki H, Nishioka K, Nakajima T (2006). Comparative analysis of gene expression profiles in intact and damaged of human osteoarthritic cartilage. Arthritis Rheum.

[71] Terkeltaub R, Johnson K, Murphy A, Ghosh S (2002). Invited review: The mitochondrion in osteoarthritis. Mitochondrion.

[72] Maneiro E, Martín MA, de Andres MC, López-Armada MJ, Fernández-Sueiro JL, del Hoyo P, Galdo F, Arenas J, Blanco FJ (2003). Mitochondrial respiratory activity is altered in osteoarthritic human articular chondrocytes. Arthritis Rheum.

[73] Henrotin Y, Blanco FJ, Aigner T, Kurz B (2007). The significance of oxidative stress in articular cartilage ageing and degradation. Curr. Rheumatol. Rev.

[74] Taylor RW, Turnbull DM (2005). Mitochondrial DNA mutations in human disease. Nat. Rev. Genet.

[75] Torroni A, Huoponen K, Francalacci P, Petrozzi M, Morelli L, Scozzari R, Obinu D, Savontaus ML, Wallace DC (1996). Classification of European mtDNAs from an analysis of three European populations. Genetics.

[76] Wallace DC, Brown MD, Melov S, Graham B, Lott M (1998). Mitochondrial biology, degenerative diseases and aging. Biofactors.

[77] Wallace DC (2005). A mitochondrial paradigm of metabolic and degenerative diseases, aging, and cancer: a dawn for evolutionary medicine. Annu. Rev. Genet.

[78] Niemi AK, Hervonen A, Hurme M, Karhunen PJ, Jylhä M, Majamaa K (2003). Mitochondrial DNA polymorphisms associated with longevity in a Finnish population. Hum. Genet.

[79] Rego I, Fernandez-Moreno M, Fernandez-Lopez C, Arenas J, Blanco FJ (2008). Mitochondrial DNA haplogroups: Role in prevalence and severity of knee osteoarthritis. Arthritis Rheum.

